# A Novel Graphitic Biochar Derived from Banana Peels for Efficient PFAS Removal: Mechanistic Insight from Integrated Experiments and DFT Calculations

**DOI:** 10.3390/toxics14030204

**Published:** 2026-02-27

**Authors:** Liu-Yi Wei, Ru-Meng Wu, Zhen-Zhu Liu, Feng-Jiao Peng, Jun-Jie Hu, Chang-Gui Pan

**Affiliations:** 1 School of Resources, Environment and Materials, Guangxi University, Nanning 530004, China; weiliuyi2023@163.com (L.-Y.W.); 2215393038@st.gxu.edu.cn (Z.-Z.L.); 2 Guangxi Laboratory on the Study of Coral Reefs in the South China Sea, School of Marine Sciences, Guangxi University, Nanning 530004, China; 19806859662@163.com (R.-M.W.); 2227402004@st.gxu.edu.cn (J.-J.H.); 3 Guangdong Provincial Key Laboratory of Chemical Pollution and Environmental Safety, MOE Key Laboratory of Theoretical Chemistry of Environment, SCNU Environmental Research Institute, South China Normal University, Guangzhou 510006, China; fengjiaopeng@m.scnu.edu.cn; 4 School of Environment, South China Normal University, Guangzhou 510006, China

**Keywords:** PFAS adsorption, aquatic remediation, banana peel, adsorption interaction, computational chemistry

## Abstract

Per- and polyfluoroalkyl substances (PFASs) have raised considerable concern due to their ubiquity, persistence, bioaccumulation, and toxicity. However, cost-effective, high-performance adsorbents for PFAS removal from aquatic environments remain limited. Here, we synthesized a porous graphitic biochar adsorbent (Zn-BBC) from banana peel waste via zinc chloride (ZnCl_2_) activation and applied it to removing ten legacy and alternative PFASs from water. Zn-BBC achieved removal efficiencies > 95% for all target PFASs. The adsorption of PFASs onto Zn-BBC followed pseudo-second-order (PSO) kinetics, suggesting chemisorption. Additionally, the adsorption isotherms were well described by the Sips model, indicating surface heterogeneity. Zn-BBC exhibited robust performance over a broad pH range (3–9). Coexisting ions (CO_3_^2−^, SO_4_^2−^, Zn^2+^, Ca^2+^, and Mg^2+^), tested individually at 10 mM each, had negligible effects on the adsorption of the PFASs examined, except for perfluorobutanoic acid (PFBA). In contrast, humic acid (10 mM) significantly reduced the removal rates of PFBA, perfluorohexanoic acid (PFHxA), and hexafluoropropylene oxide dimer acid (GenX). Nevertheless, in river and lake waters, Zn-BBC achieved >85.0% removal of all PFASs except PFBA. In regeneration experiments, Zn-BBC exhibited excellent reusability. Experimental characterization and density functional theory (DFT) calculations jointly revealed that PFAS adsorption involves electrostatic interactions, hydrophobic interactions, π-CF interactions, surface complexation, and hydrogen bonding. These results suggest that Zn-BBC is a promising sorbent for PFAS removal in water.

## 1. Introduction

Per- and polyfluoroalkyl substances (PFAS) are synthetic organofluorine compounds characterized by fully or partially fluorinated carbon chains and diverse chain lengths and functional groups [1,2]. Because of their hydrophobic and oleophobic properties and high chemical and thermal stability, PFASs are extensively used in a variety of commercial and industrial applications, such as textiles, firefighting foams, pesticides, and food packaging [1,3,4]. Correspondingly, PFAS residues are widely detected worldwide, particularly in aquatic environments [5]. However, PFAS exposure may pose potential risks to human health, including hepatotoxicity, reproductive toxicity, carcinogenicity, and endocrine disruption [6,7]. Therefore, the use of two representative PFASs, perfluorooctane sulfonate (PFOS) and perfluorooctanoic acid (PFOA), has been restricted under the Stockholm Convention since 2009 and 2019, respectively. Furthermore, on 10 April 2024, the U.S. Environmental Protection Agency (EPA) finalized drinking water standards, setting legally enforceable Maximum Contaminant Levels (MCLs) of 4 ng/L each for both PFOS and PFOA. In response to the phase-out of legacy PFASs, numerous alternatives have been introduced to the market [8], such as hexafluoropropylene oxide dimer acid (GenX), 6:2 fluorotonic carboxylic acid (6:2 FTSA), and 6:2 fluorotelomer sulfonic acid (6:2 FTCA). However, these alternatives have also been demonstrated to pose risks to human and ecosystem health [9,10] Therefore, it is crucial to develop effective technologies to mitigate pollution from both legacy and alternative PFASs in aquatic environments.

Adsorption is among the most effective PFAS-removal methods for water because it is cost-effective, simple to operate, and highly efficient [5]. Over the past several decades, a broad range of adsorbents have been developed, including ion exchange resins [11], activated carbon [12], modified clays [13], molecularly imprinted polymers [14], metal–organic frameworks (MOFs) [15], and biochar [5]. Notably, biochar has emerged as a promising adsorbent for PFAS removal from the aqueous phase owing to its high surface area, diverse pore structure, and ease of preparation [16]. However, pristine biochar generally shows limited removal efficiency for organic contaminants [17,18]. For instance, the removal efficiencies for PFOS, PFOA, perfluorobutanoic acid (PFBA), and perfluorobutane sulfonic acid (PFBS) using sawdust-derived biochar ranged from 25% to 42% [19]. To enhance the adsorption performance of pristine biochar, various modifications have been investigated, including acid treatment [20], alkaline treatment [21], and metal impregnation [22]. Among these modifications, metal impregnation is the most widely used, owing to its ease of preparation and the high adsorption capacity of the resulting biochar [23]. For instance, the adsorption capacity of sludge-derived biochar for PFOA increased from 194.00 μmol/g to 469.65 μmol/g after impregnation with ferric chloride [24]. Likewise, zinc chloride (ZnCl_2_)-modified biochar yielded enhanced adsorption of ciprofloxacin and hexavalent chromium [25,26]. In particular, ZnCl_2_-modified Spartina alterniflora-derived biochar showed more than a tenfold increase in adsorption capacity for both sulfadiazine (SDZ) and ciprofloxacin (CIP) [27]. More recently, this modification strategy has been extended to PFAS remediation. Afrooz et al. demonstrated that ZnCl_2_-activated canola straw biochar achieved high adsorption capacities for both PFOA and PFOS [28]. These findings motivate the hypothesis that ZnCl_2_ modification can improve PFAS sorption by promoting pore formation and graphitic/aromatic structures and by introducing Zn-associated surface sites that strengthen specific interfacial interactions.

Elucidating the adsorption mechanisms at the molecular level is paramount for the rational design of advanced adsorbents. Density Functional Theory (DFT) calculations provide molecular-level insight into adsorption configurations and energetics, enabling the identification of dominant interactions (e.g., adsorption energy, hydrogen bonding, and dispersion force) [29], and the role of surface functional groups in stabilizing PFAS at sorbent interfaces [30]. DFT has been extensively employed to elucidate interactions with carbonaceous surfaces, such as electrostatic attraction and complexation with metal sites [31,32], thereby bridging macroscopic observations and molecular mechanisms. Accordingly, integrating batch adsorption experiments with DFT can move beyond empirical performance comparisons and directly test structure–function relationships for modified biochar.

To align with circular economy principles and reduce cost, we selected banana peel as the feedstock. Bananas are among the most widely consumed fruits worldwide. Banana peels are commonly used as animal feed and as biofertilizers [33]. However, less than 10% of banana peels are productively utilized, with most being discarded, resulting in substantial biomass waste and exacerbating environmental pollution [34]. Beyond availability, banana peels are a chemically favorable biochar precursor because their lignocellulosic matrix and oxygen-containing functional groups (e.g., hydroxyl and carboxyl moieties) can translate into abundant surface functionalities after pyrolysis [35,36], while their inherent biomass architecture can support the formation of hierarchical pore structures during chemical activation [37]. Converting banana peels into biochar adsorbents via pyrolysis is a practical valorization route that facilitates resource recovery and environmental impact mitigation. For example, banana peel biochar could reach adsorption capacities of up to ~1790 mg/g for malachite green (MG) under specific experimental conditions [34]. Similarly, the adsorption capacity of banana peel biochar for methylene blue (MB) substantially increased from 40.19 to 83.56 mg/g following iron-oxide modification [38]. However, to date, banana peel biochar has only been used for adsorption-based removal of a limited range of pollutants (e.g., MG, MB, and tetracycline) from the aqueous phase [20,34,38]. To the best of our knowledge, banana peel biochar has not yet been applied to PFAS removal, and the underlying adsorption mechanisms remain unclear.

The overarching aim of this study is to develop a cost-effective, waste-derived adsorbent for efficient PFAS removal and to elucidate the underlying mechanisms. By transforming banana peel waste into an effective PFAS adsorbent, this work contributes to multiple United Nations Sustainable Development Goals (SDGs), particularly SDG 6 (Clean Water and Sanitation) through improved removal of persistent contaminants, SDG 12 (Responsible Consumption and Production) via waste-to-resource valorization of banana peels, and SDG 3 (Good Health and Well-Being) by reducing PFAS exposure risks. Furthermore, the conversion of biomass waste into carbon materials may offer broader sustainability co-benefits relevant to SDG 13 (Climate Action).

The objectives of this study were to (i) synthesize and characterize a ZnCl_2_-modified biochar derived from banana peels (Zn-BBC); (ii) investigate the adsorption performance of Zn-BBC for PFASs and evaluate the effects of pH, coexisting ions, and humic acid (HA) on removal efficiency; (iii) elucidate the adsorption mechanisms of PFASs on Zn-BBC using both experimental and theoretical approaches; and (iv) evaluate the practical applicability of Zn-BBC via regeneration experiments.

## 2. Materials and Methods

### 2.1. Standards and Reagents

This study selected ten PFASs as target compounds: seven legacy PFASs (i.e., PFOS, PFOA, PFBA, PFBS, perfluorohexanoic acid (PFHxA), perfluoroheptanoic acid (PFHpA), and perfluorohexane sulfonic acid (PFHxS); Wellington Laboratories, Guelph, ON, Canada) and three alternatives (i.e., 6:2 FTSA, 6:2 FTCA, and GenX; International Laboratory USA, San Francisco, CA, USA). Details of the individual PFASs are provided in Tables S1 and S2, and reagent information is given in Text S1.

### 2.2. Biochar Preparation and Characterization

Banana peels were oven-dried at 105 °C for 48 h, ground with a small grinder, and sieved through an 80-mesh screen to obtain a uniform powder. Then, 2 g of banana peel powder and 2 g of ZnCl_2_ (CNW, ANPEL Laboratory Technologies Inc., Shanghai, China) were placed into a mortar and ground for 3 min before being transferred to a crucible. The crucible was then heated to 500 °C at 10 °C/min in a tube furnace under nitrogen with a flow rate of 80 sccm, and held for 2 h. Thereafter, the biochar was cooled to room temperature, washed several times with 0.1 mol/L HCl (CNW, ANPEL Laboratory Technologies Inc., Shanghai, China) and deionized water until the rinse water reached pH 7.0, oven-dried (WP-25A, Tianjin Taiste Instrument Co., Ltd., Tianjin, China) at 105 °C for 6 h, and labeled Zn-BBC (ZnCl_2_-modified banana peel biochar). Pristine banana peel biochar, prepared following the same protocol but omitting ZnCl_2_, was designated BBC. The synthesis of Zn-BBC and BBC is shown in Figure S1.

BBC and Zn-BBC were characterized before and after PFAS adsorption using scanning electron microscopy (SEM, SU5000, Hitachi, Tokyo, Japan), energy dispersive X-ray spectroscopy (EDS coupled with SEM), X-ray powder diffraction (XRD, D8 Discover, Bruker, Karlsruhe, Germany), Raman spectroscopy (InVia Qontor, Renishaw, Wotton-under-Edge, UK), Fourier transform infrared spectroscopy (FTIR, IRTrace-100, SHIMADZU, Kyoto, Japan), X-ray photoelectron spectroscopy (XPS, Nexsa, Thermo Fisher, Waltham, MA, USA), Brunauer–Emmett–Teller (BET, Autosorb-iQ, Quantachrome, Boynton Beach, FL, USA), particle size distribution analysis (Mastersizer 2000, Malvern Instruments, Malvern, UK) and a zeta potential measurement instrument (Zetasizer Nano ZS90, Malvern Instruments, Malvern, UK). To probe adsorption mechanisms, the modified biochar was equilibrated with mixed-PFAS solution at the highest tested concentration, and the resulting spent biochar was then analyzed. Details of the biochar characterization are provided in Text S2. The particle size data (μm) for BBC and Zn-BBC are presented in Table S5.

### 2.3. Batch Adsorption Experiment

The adsorption performance for PFASs of BBC and Zn-BBC was evaluated. Specifically, 40 mL of ultrapure water and 10 mg of Zn-BBC or BBC were added to a 50 mL centrifuge tube, resulting in an initial concentration of 100 μg/L for each PFAS. Unless otherwise specified, all batch adsorption experiments were conducted at 25 °C and pH 7.0 (adjusted with 0.1 mol/L HCl or NaOH (CNW, ANPEL Laboratory Technologies Inc., Shanghai, China)). For adsorption kinetics, the mixtures were shaken on an orbital shaker (IS-RDD3, Suzhou Jiemei Electronics Co., Ltd., Suzhou, Jiangsu, China) at 300 rpm under room temperature. Then, 200 µL aliquots were withdrawn at specified time points (0, 5, 10, 15, 30, 60, 120, 240, 480, 720, and 1440 min), transferred to 0.5 mL centrifuge tubes, and centrifuged (3K15, Sigma Laborzentrifugen GmbH, Osterode, Germany) at 13,000 rpm for 5 min. An aliquot of 100 μL of the supernatant was then transferred to a 2 mL centrifuge tube, mixed with 400 μL of methanol (HPLC-grade, Merck Corporation, Darmstadt, Germany), filtered through a 0.22 μm filter membrane (organic phase, ANPEL, Shanghai, China), and finally transferred to a polypropylene vial for PFAS analysis. For adsorption isotherms, 10 mg of Zn-BBC was contacted with 40 mL of the PFAS mixture, with each PFAS at an initial concentration of 100, 200, 500, 800, 1000, and 2000 μg/L and the subsequent steps were the same as those in the adsorption kinetics.

The effects of pH, coexisting ions, and HA on PFAS adsorption were also examined (an initial concentration of 100 μg/L for each PFAS). Specifically, pH values of 3, 5, 7, 9, and 11 were tested, adjusted with 0.1 mol/L NaOH and 0.1 mol/L HCl; and 10 mM CO_3_^2−^, SO_4_^2−^, Zn^2+^, Ca^2+^, Mg^2+^(CNW, ANPEL Laboratory Technologies Inc., Shanghai, China), and HA (Alfa Aesar, Shanghai, China) were tested separately. Additionally, 40 mL aliquots of raw river water (pH 7.85) and lake water (pH 8.20) were tested separately. All the above experiments were conducted for 1440 min, with all other steps identical to those of the adsorption kinetics. After PFAS adsorption, Zn-BBC was subjected to five consecutive adsorption–desorption cycles using methanol as the eluent solvent. Each experiment was conducted in triplicate.

### 2.4. Statistical Analysis

All batch adsorption experiments were performed in triplicate, and the results are expressed as mean ± standard deviation (SD). To evaluate the statistical significance of the differences in PFAS removal efficiencies under varying pH conditions, multiple independent-samples *t*-tests were conducted using IBM SPSS Statistics (version 26, IBM Corp., Armonk, NY, USA). Differences were considered statistically significant at *p* < 0.05.

### 2.5. PFAS Determination and Data Analysis

PFAS concentrations were determined using ultra-high-performance liquid chromatography with triple quadrupole mass spectrometry (UPLC-MS/MS, 1290-6460, Agilent Technologies, Santa Clara, CA, USA). Detailed information on the instrumental methods is shown in Tables S1, S3 and S4. Additionally, details of the data analysis for removal efficiency, adsorption capacity, adsorption kinetics, and adsorption isotherms are provided in Text S3.

### 2.6. Density Functional Theory (DFT) Calculations

To simplify the computations, we modeled the pristine Zn-BBC biochar surface using a heptagonal graphene structure [39]. Based on XPS and FTIR analyses, we constructed a structural model of Zn-BBC by incorporating the corresponding surface functional groups (i.e., O-C=O, C-O, -OH, O-Zn, and C=O). Because of the high structural similarity among the target PFASs, PFBA and PFOS were selected as representative compounds for adsorption-energy calculations. The molecular models were formulated using Gaussian 16 (Gaussian, Inc., Wallingford, CT, USA), and adsorption energies were calculated using DFT. The B3LYP functional and the 6-31G (d,p) basis set were used for all calculations. Solvation energies were computed based on the SMD model using water as the solvent to match experimental conditions [40]. Dispersion corrections (DFT-D3) were applied to improve the calculation accuracy. The energy gap (E_g_) was defined as the difference between the energy of the highest occupied molecular orbital (HOMO) of the adsorbent and the energy of the lowest unoccupied molecular orbital (LUMO) of the adsorbate molecule. The adsorption energy was calculated using the formula listed below:

E_bind_ = E_complex_ − (E_Zn-BBC_ + E_PFAS_) (1)

where E_bind_ and E_complex_ denote the adsorption energy and total energy of the Zn-BBC and PFAS complex, respectively. E_Zn-BBC_ and E_PFAS_ are the total energies of the Zn-BBC and PFAS congeners, respectively.

## 3. Results and Discussion

### 3.1. Characterization of BBC and Zn-BBC

**Figure 1:**
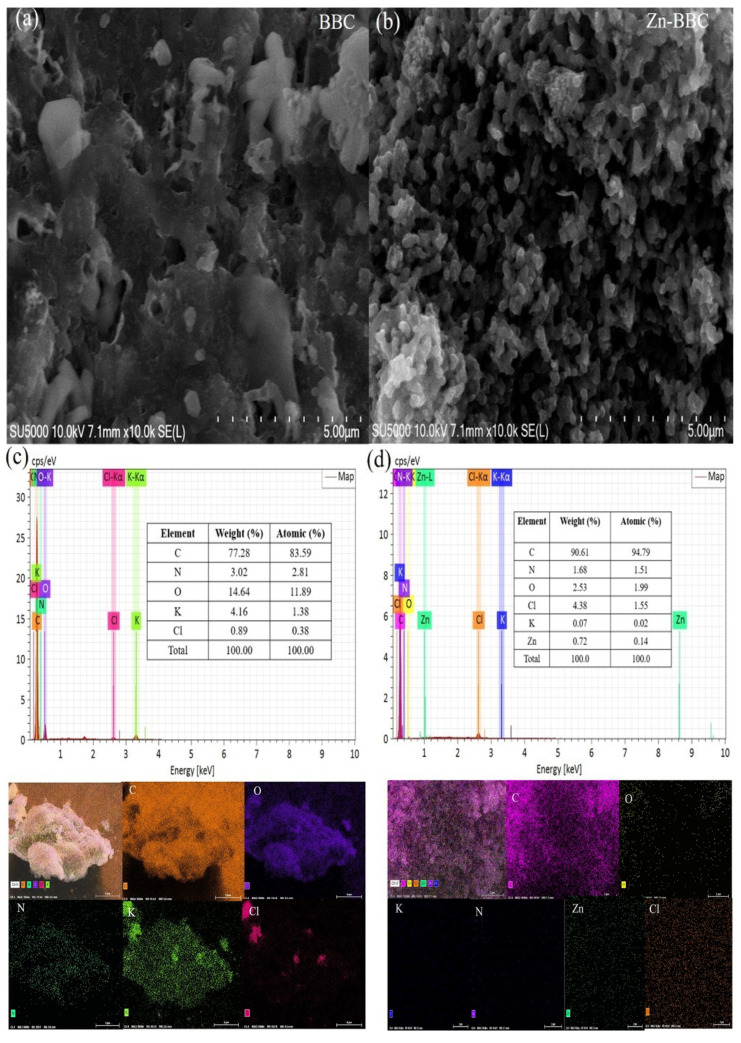
Scanning electron microscopy (SEM) images of BBC (**a**) and Zn-BBC (**b**), and energy dispersive X-ray spectroscopy (EDS) spectra of BBC (**c**) and Zn-BBC (**d**).

SEM images revealed marked differences in surface morphology between BBC and Zn-BBC (Figure 1a,b). BBC showed few discernible pores, whereas Zn-BBC exhibited an abundantly porous surface. This difference is likely due to ZnCl_2_-assisted activation during pyrolysis: molten ZnCl_2_ promotes dehydration/aromatization, and subsequent acid washing removes Zn-containing residues, thereby generating additional pores in Zn-BBC [25]. After modification, the specific surface area, average pore diameter, and pore volume of Zn-BBC sharply increased from 34 m^2^/g to 1157 m^2^/g, 3.41 nm to 3.82 nm, and 0.05 cm^3^/g to 0.33 cm^3^/g, respectively. Additionally, the particle size range shifted from 19.33–287.75 μm to 47.16–198.03 μm (Table S5). Based on the N_2_ adsorption–desorption data, both BBC and Zn-BBC exhibited type IV isotherms with an H4 hysteresis loop (Figure S2a,b), indicating mesoporosity [41]. This was further confirmed by the pore-size distribution analyses (Figure S2c,d). The presence of elements (e.g., C, O, N, Cl, and K) in BBC was identified with EDS (Figure 1c). Following ZnCl_2_ pyrolysis, the C content increased from 77.28% to 90.61%, and Zn was incorporated at 0.72 wt% (Figure 1d), confirming successful modification.

**Figure 2:**
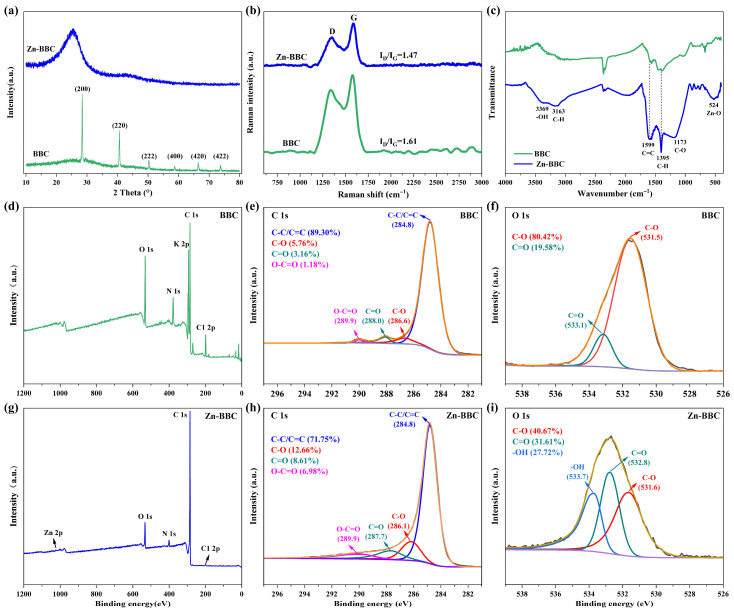
X-ray powder diffraction (XRD) patterns (**a**), Raman spectra (**b**), and Fourier transform infrared spectroscopy (FTIR) spectra (**c**) for BBC and Zn-BBC. Overall X-ray photoelectron spectroscopy (XPS) spectra (**d**,**g**) and C 1s (**e**,**h**) and O 1s (**f**,**i**) spectra for BBC (**d**–**f**) and Zn-BBC (**g**–**i**).

The XRD pattern of BBC revealed that peaks occurred at 2θ = 28.44°, 40.62°, 50.22°, 58.78°, 66.42°, and 73.72° (Figure 2a), which could be attributed to the (200), (220), (222), (400), (420), and (422) crystal planes of KCl, respectively [34]. Compared with BBC, Zn-BBC exhibited different diffraction peaks at 23° and 42.5°, demonstrating the formation of graphitic structure (002) and crystalline carbon structure (100) [42]. In addition, Raman spectra showed distinct D and G bands between BBC and Zn-BBC, and the ID/IG ratio of Zn-BBC (1.47) was lower than that of BBC (1.61; Figure 2b), indicating a higher degree of graphitization for Zn-BBC [41].

FTIR analysis showed a band at ~3369 cm^−1^ (Figure 2c), assigned to the O-H stretching vibration of surface hydroxyl groups (-OH) [22]. In addition, the bands at ~1599 and ~1173 cm^−1^ were assigned to aromatic C=C stretching and C-O stretching, respectively [43], suggesting the formation of highly stable aromatic structures in Zn-BBC. Additional bands at ~1395 and ~524 cm^−1^ were also observed in Zn-BBC and assigned to C-H bending and Zn-O stretching, respectively [44,45].

XPS spectra of BBC and Zn-BBC are shown in Figure 2d–i. The C 1s peaks of both biochars were assigned to C-C/C=C (284.8 eV), C-O (286.1–286.6 eV), C=O (287.7–288.0 eV), and O-C=O (289.9 eV) [46]. C-C/C=C was the predominant form of C 1s in both BBC and Zn-BBC, accounting for 89.30% and 71.75% of the C 1s signal, respectively. Following ZnCl_2_ modification, the relative fractions of C-O and C=O increased from 5.76% to 12.66% and 3.16% to 8.61%, respectively, suggesting an increase in oxygen-containing functional groups. The O 1s peaks were assigned to C-O (531.5 eV) and C=O (533.1 eV) for BBC, and to C-O (531.6 eV), C=O (532.8 eV), and -OH (533.7 eV) for Zn-BBC [21]. Additionally, a weak Zn 2p signal was observed for Zn-BBC (Figure S3), supporting the presence of trace Zn species retained after ZnCl_2_-assisted activation and washing.

### 3.2. Adsorption Kinetic and Isotherms

Removal efficiencies across the tested PFASs were 8.0–68.6% for BBC and 95.7–100% for Zn-BBC (Figure S4). The superior performance of Zn-BBC is consistent with its higher specific surface area and more developed pore structure, which provide more adsorption sites and markedly enhance adsorption [41]. In comparison with adsorbents reported in the literature (Table S6), Zn-BBC reached adsorption equilibrium more rapidly at comparable initial PFAS concentrations. These results indicate that ZnCl_2_ modification substantially enhanced PFAS adsorption performance. Zn-BBC was thus used for all subsequent experiments.

The adsorption kinetics of the PFASs are shown in Figure S5. Adsorption was rapid in the first 5 min (>90% removal for several PFASs) and then slowed, reaching equilibrium within 30 min. The rapid initial adsorption of PFAS can be ascribed to the high density of accessible active sites on the Zn-BBC surface. Across the tested PFASs, the pseudo-second-order (PSO) model fit better (higher R^2^: 0.993–0.999 vs. 0.977–0.995) and yielded Q_e_ estimates more consistent with experiment than the pseudo-first-order (PFO) model (Table S7). These results indicate that chemisorption contributed substantially to the adsorption of PFASs [34].

Further investigation of adsorption isotherms was performed to elucidate interactions between the PFASs and Zn-BBC (Figure S6). For PFHxA, the R^2^ values of the Freundlich model (0.98) and the Sips model (0.96) were both lower than that of the Langmuir model (0.99; Table S8), indicating monolayer adsorption of PFHxA onto Zn-BBC [47]. For the remaining nine PFASs, the R^2^ values of the Sips model (0.93–0.99) were greater than those of the Freundlich model (0.52–0.99) and the Langmuir model (0.62–0.98). In the Sips isotherm, the heterogeneity index m (0.37–3.08) deviated markedly from unity, indicating a heterogeneous adsorption process [48]. Accordingly, heterogeneous models (Sips/Freundlich) describe the interactions between these PFASs and Zn-BBC better than Langmuir. Maximum adsorption capacities were obtained by isotherm fitting: Langmuir for PFHxA and Sips for the remaining nine PFASs. As expected, the equilibrium adsorption amount (q_e_) increased with the initial concentration (Figure S6). The fitted adsorption capacities ranged from 6.07 mg/g for PFBA to 9.41 mg/g for PFOS.

### 3.3. The Effects of pH, Coexisting Ions, and HA on PFAS Removal

**Figure 3:**
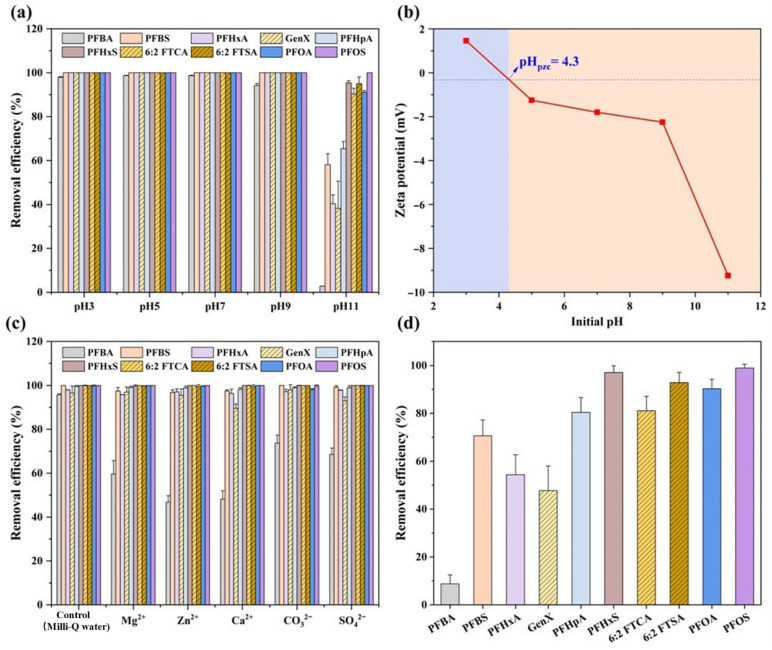
PFAS removal efficiency by Zn-BBC across pH 3–11 (**a**); Zeta potential of Zn-BBC as a function of pH (**b**); PFAS removal efficiency by Zn-BBC in the presence of 10 mM coexisting ions (**c**) and 10 mM humic acid (**d**).

For clarity, in this study, perfluorocarboxylic acids (PFCAs) with chain lengths of C4-C6 and perfluorosulfonic acids (PFSAs) with chain lengths ≤ C5 are classified as ‘short-chain’ PFASs (PFBA, PFBS, PFHxA, GenX). Their chain lengths ≥ C7 (for PFCAs) and ≥C6 (for PFSAs) are termed ‘long-chain’ (PFHpA, PFOA, PFHxS, PFOS, 6:2 FTCA, 6:2 FTSA) [49]. PFAS removal efficiencies using Zn-BBC across pH 3–11 are presented in Figure 3a. Over the pH range 3–9, removal efficiencies were ≥94.2% for all PFASs tested. At pH 11, however, removal efficiencies decreased sharply for all short-chain PFASs (*p* < 0.05): PFBA fell from ≥94.2% to 2.77%, and PFBS, PFHxA, and GenX declined from 100% to 58.1%, 40.4%, and 38.2%, respectively. In contrast, removal efficiencies were still >90% for all long-chain PFASs, except for PFHpA (65.4%). All examined PFASs exist predominantly as anions over pH 3–11, given pKa values well below this range (−3.27 to 2.82; Table S2). Therefore, the near-constant removal across pH 3–9 likely reflects the dominance of hydrophobic interactions over electrostatic interactions. Meanwhile, the reduced removal of short-chain PFASs (especially PFBA) at pH 11 is likely due to the enhanced electrostatic repulsion caused by the more negative zeta-potential of Zn-BBC [50], dropping from −2.25 mV at pH 9 to −9.24 mV at pH 11 (Figure 3b). This can be analyzed by considering the competing roles of hydrophobic attraction and electrostatic repulsion. PFBA with the shortest carbon chain (C3) exhibits the weakest hydrophobic interaction. The intensified electrostatic repulsion easily overwhelmed this weak hydrophobic attraction. In contrast, slightly longer short-chain PFASs (e.g., PFBS, C4) retain marginally stronger hydrophobic interactions, allowing for partial adsorption despite the repulsion. In contrast to short-chain PFASs, long-chain PFAS engage in hydrophobic interactions strong enough to overcome electrostatic repulsion [12], enabling stable adsorption onto Zn-BBC under strongly alkaline conditions (pH 11).

PFAS removal efficiencies by Zn-BBC with coexisting ions (CO_3_^2−^, SO_4_^2−^, Zn^2+^, Ca^2+^, and Mg^2+^) present are shown in Figure 3c. The presence of coexisting ions (10 mM each) had little effect on PFAS removal, except for PFBA, for which removal efficiency decreased from 95.7% to 46.9–73.7%. Among the target PFASs, PFBA is the most water-soluble and least hydrophobic [51], resulting in weak hydrophobic/van der Waals interactions with the adsorbent. In the presence of coexisting ions, PFBA adsorption was further diminished owing to site competition and screening of electrostatic attractions [52]. Similar results have been reported for this PFAS in the presence of Na^+^, Cl^−^, SO_4_^2−^, NO_3_^−^, and PO_4_^3−^ [53,54,55]. For example, the removal efficiency of PFBA by quaternary ammonium-functionalized nanocellulose dropped to 0% in the presence of 0.1 M NaCl [54].

In the presence of HA, removal efficiencies decreased for all tested PFASs by 1.10–87.0%, depending on the compound (Figure 3d). Nevertheless, removal efficiencies were still above 80% for the long-chain PFASs. The lower PFAS removal efficiencies, relative to water-only systems, could be attributed to two factors. First, HA adsorbs onto Zn-BBC and, via its deprotonated carboxylate and phenolate groups [52], increases the negative surface charge, thereby enhancing electrostatic repulsion between the HA-coated sorbent and PFASs and reducing adsorption [56]. Second, competitive adsorption by HA leads to pore blockage and site occupation on Zn-BBC, thereby decreasing the number of accessible sites for PFASs [57]. Moreover, hydrophobic domains in HA can self-associate via hydrophobic interactions, forming clusters that deposit on Zn-BBC and block its pore structure [19].

### 3.4. Adsorption Mechanisms

**Figure 4:**
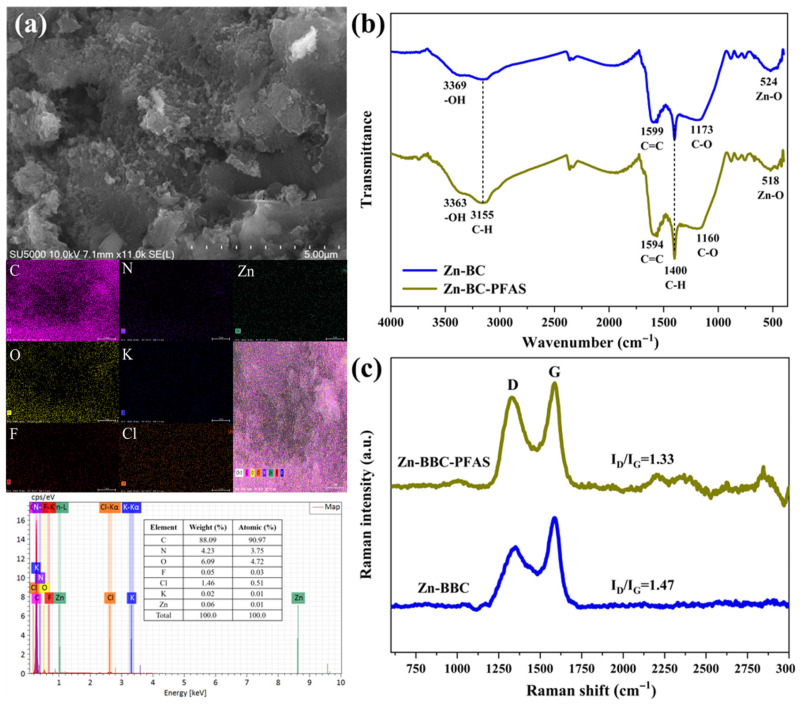
Scanning electron microscopy (SEM) and energy dispersive X-ray spectroscopy (EDS) images of Zn-BBC-PFAS (**a**); Fourier transform infrared spectroscopy (FTIR) spectra (**b**) and Raman spectra (**c**) for Zn-BBC and Zn-BBC-PFAS.

Multiple mechanisms contributed to the adsorption of PFAS onto Zn-BBC. SEM images of Zn-BBC after adsorption showed pore infilling and flocculent aggregates, with an apparent reduction in visible surface porosity (Figure 4a), providing evidence of PFAS adsorption. EDS mapping further detected fluorine uniformly distributed across the Zn-BBC surface (Figure 4a), supporting the presence of PFAS on/within the sorbent [58]. EDS semi-quantification showed a decrease in Cl content from 4.38% to 1.46% and a slight increase in F content (0.05%) after adsorption, consistent with partial displacement of residual chloride and adsorption of PFAS (Figure 4a). Correspondingly, changes in the XPS Cl 2p and F 1s signals (Figure S7), together with FTIR band shifts of 5–13 cm^−1^ (Figure 4b), indicate contributions from electrostatic interactions and specific binding (e.g., hydrogen bonding/complexation), pointing to multiple adsorption mechanisms rather than a single one [52,59,60,61].

**Figure 5:**
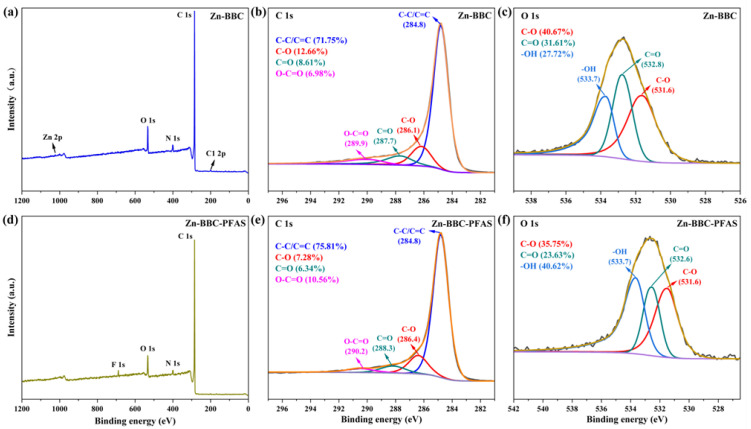
Overall X-ray photoelectron spectroscopy (XPS) spectra (**a**,**d**) and C 1s (**b**,**e**) and O 1s (**c**,**f**) spectra for Zn-BBC (**a**–**c**) and Zn-BBC-PFAS (**d**–**f**).

FTIR spectra showed that the aromatic C=C band shifted from 1599 to 1594 cm^−1^ and increased in intensity upon PFAS adsorption (Figure 4b), indicating the involvement of π-CF interactions [62]. Likewise, Raman spectra showed a decrease in the I_D_/I_G_ ratio from 1.47 to 1.33 after PFAS adsorption (Figure 4c), suggesting π-CF interactions between PFAS C-F bonds and the graphitic domains of Zn-BBC [34]. Evidence for π-CF interactions was further provided by XPS C 1s spectral deconvolution [63], which showed an increase in the C-C/C=C component of Zn-BBC from 71.75% to 75.81% after PFAS adsorption (Figure 5). Here, Zn-BBC is a highly graphitized biochar and therefore possesses extensive sp^2^ (C=C) domains [64]. Given that sp^2^ content correlates positively with electron-accepting capacity [64], Zn-BBC can act as a π-electron acceptor [65], thereby enhancing its interactions with amphiphilic PFAS.

After PFAS adsorption, the Zn-O stretching band at ~524 cm^−1^ decreased in intensity and showed a red shift to 518 cm^−1^ (Figure 4b). These spectral changes are consistent with Zn-O involvement in adsorption via surface complexation with PFASs [66]. The O-H stretching band in the FTIR spectra shifted from 3369 to 3363 cm^−1^ and increased in intensity upon PFAS adsorption (Figure 4b), indicating hydrogen bonding of surface hydroxyls with PFASs [67]. Deconvolution of the XPS C 1s spectra showed decreased relative contributions of the C-O (from 12.66% to 7.28%) and C=O (from 8.61% to 6.34%) components after adsorption (Figure 5). The O 1s spectra corroborated this trend, exhibiting concomitant declines in the C-O (from 40.67% to 35.75%) and C=O peaks (from 31.61% to 23.63%) (Figure 5). Taken together, these results support the participation of oxygen-containing groups (C=O and C-O) in PFAS binding via hydrogen bonding [63]. Furthermore, the greater adsorption affinity of long-chain PFASs relative to short-chain analogues indicates an additional contribution from hydrophobic interactions [54].

**Figure 6:**
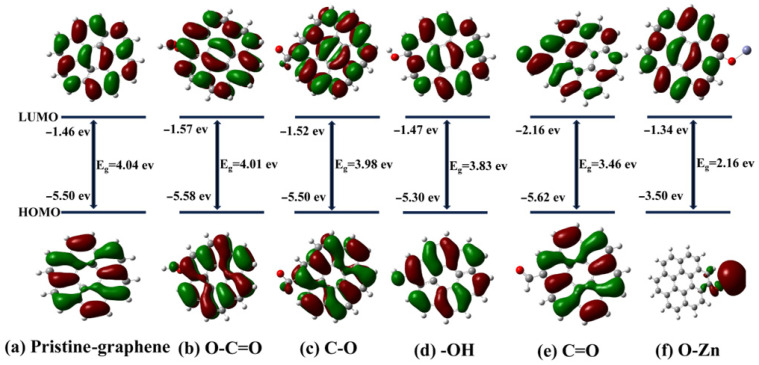
HOMO/LUMO energy levels for pristine graphene (**a**) and its functionalized derivatives bearing O-C=O (**b**), C-O (**c**), -OH (**d**), C=O (**e**), and O-Zn (**f**) groups.

HOMO-LUMO energy gaps (E_g_) were computed for graphene clusters functionalized with C-O, O-Zn, O-C=O, -OH, and C=O to elucidate PFAS adsorption on Zn-BBC (Figure 6). Larger E_g_ values generally indicate lower electronic reactivity (greater kinetic stability) [56,68]. The calculated E_g_ of the pristine graphene cluster was 4.04 eV and decreased to 4.01, 3.98, 3.83, 3.46, and 2.16 eV upon functionalization with O-C=O, C-O, -OH, C=O, and O-Zn, respectively. The reduced gaps imply enhanced electronic reactivity and a greater propensity for charge transfer [40]. The O-Zn-functionalized cluster exhibited the smallest E_g_, suggesting it most readily engages in charge transfer, thereby facilitating adsorption of PFAS molecules.

**Figure 7:**
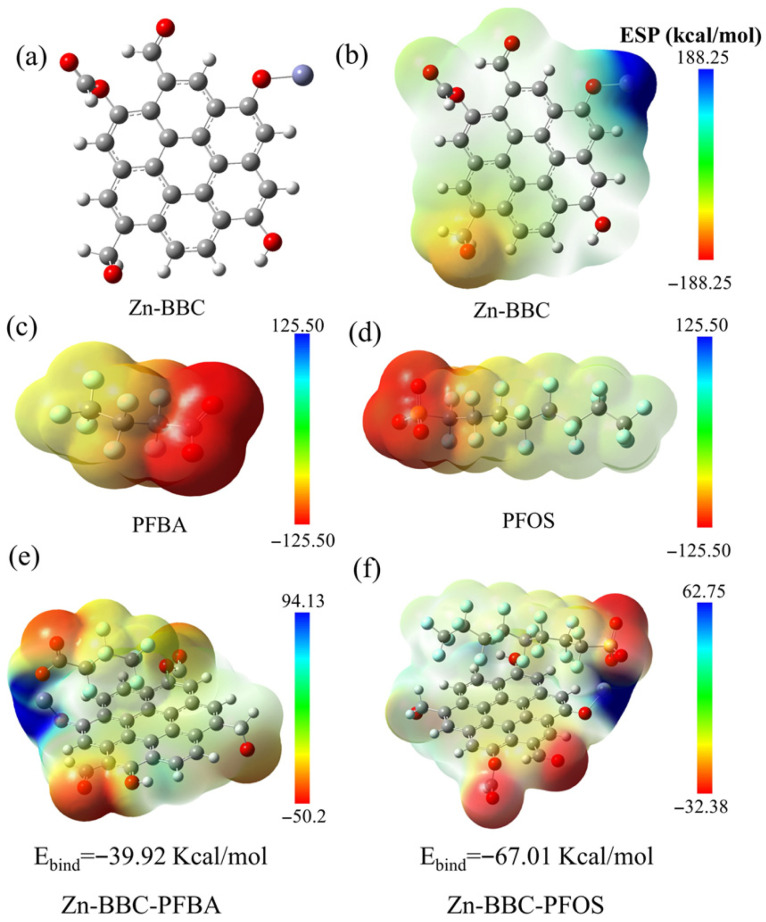
Molecular structure (**a**) and electrostatic potential map (**b**) of Zn-BBC. Electrostatic potential of PFBA (**c**) and PFOS (**d**) in the ionic state. Electrostatic potential and adsorption energy of PFBA (**e**) and PFOS (**f**) on Zn-BBC. In the molecular structure, gray spheres represent carbon atoms (C), red spheres represent oxygen atoms (O), blue spheres represent fluorine atoms (F), and white spheres represent hydrogen atoms (H).

Electrostatic potential (ESP) maps of Zn-BBC show the most positive region localized at the Zn center of the Zn-O moiety (Figure 7a,b). The anionic headgroups of PFOS (-SO_3_^−^) and PFBA (-COO^−^) are therefore electrostatically attracted to Zn, consistent with surface complexation, while the perfluoroalkyl tails interact with aromatic domains in Zn-BBC via π-CF interactions (Figure 7c,d). To quantify interaction strength, DFT calculations yielded negative adsorption energies for both PFASs (−67.01 and −39.92 kcal/mol for PFOS and PFBA, respectively; Figure 7e,f). This result indicates that Zn-BBC interacts more strongly with PFOS than with PFBA, consistent with experimental data showing maximal adsorption for PFOS and minimal for PFBA.

### 3.5. Applications to Real Water Samples and Regeneration/Disposal of Zn-BBC

**Figure 8:**
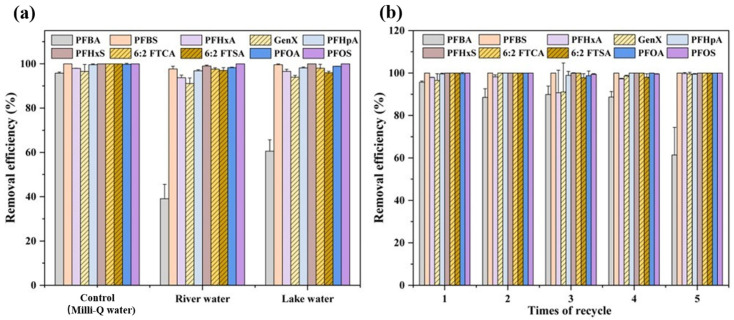
PFAS removal efficiency by Zn-BBC in different water samples (**a**). Regenerability of Zn-BBC for PFAS removal (**b**).

River and lake water were used to evaluate the applicability of Zn-BBC for PFAS removal. In lake water, removal efficiencies ranged from 60.6% to 100%; in river water, from 39.1% to 99.8% (Figure 8a). These values were slightly lower than in ultrapure water (95.7–100%). We speculate that the lower removal efficiency observed in river water may be attributed to two possible factors: a higher concentration of competing organic matter compared to lake water, as well as the influence of coexisting ions [15]. The higher pH of the South Lake (8.20) compared to the Yongjiang River (7.85) would be expected to induce stronger electrostatic repulsion and thus a greater suppression of PFAS adsorption under alkaline conditions. We therefore infer that the higher concentration of dissolved solutes, including both coexisting ions [52] and organic matter, may further exacerbate the suppression of PFBA adsorption in the Yongjiang River. Notably, Zn-BBC achieved >85.0% removal of all PFASs except PFBA in both matrices, indicating strong potential for PFAS removal from natural waters.

From economic and environmental perspectives, the recycling and regeneration of adsorbents are crucial for material competitiveness [69]. After five consecutive adsorption–desorption cycles, Zn-BBC retained removal efficiencies of >60% for PFBA and >90% for the remaining nine PFASs (Figure 8b), indicating strong regenerability and reusability and supporting its potential cost-effectiveness. After PFAS adsorption, the spent Zn-BBC can be managed by high-temperature incineration followed by stabilization/solidification. The incinerated residue is uniformly blended into a cementitious matrix (composed of cement, lime, and water) to achieve microencapsulation. After curing, the stabilized monolith can be safely landfilled [70].

## 4. Conclusions

We synthesized a ZnCl_2_-modified banana peel biochar (Zn-BBC) via one-step pyrolysis for PFAS removal in water. Zn-BBC achieved satisfactory removal efficiencies for all target PFAS except PFBA across pH 3–9, in the presence of common anions/cations and HA, and in real water samples. Experimental characterization together with DFT calculations show that Zn-BBC adsorbed PFASs via multiple mechanisms, including electrostatic interactions, π-CF interactions, surface complexation, hydrogen bonding, and hydrophobicity. After five consecutive adsorption–desorption cycles, Zn-BBC still retained excellent adsorption performance. Collectively, these results demonstrate that Zn-BBC is a promising adsorbent for removing PFASs from natural waters. To overcome the limited removal of short-chain PFASs (particularly PFBA) under environmentally relevant conditions, future efforts should focus on rationally designing Zn-BBC surface chemistry with fluoroalkyl moieties and dual-functional sites.

## Data Availability

The data presented in this study are available on request from the corresponding author.

## Supplementary Materials

The following supporting information can be downloaded at: https://www.mdpi.com/article/10.3390/toxics14030204/s1, Text S1. Standards and reagents; Text S2. Biochar characterization; Text S3. Data analysis; Figure S1. Preparation flow chart for banana peel-derived biochar (BBC) and ZnCl_2_-modified banana peel-derived biochar (Zn-BBC); Figure S2. N_2_ adsorption–desorption isotherms for BBC (a) and Zn-BBC (b); Pore size analysis of BBC (c) and Zn-BBC (d); Figure S3. Zn 2p of Zn-BBC revealed by XPS spectra. Figure S4. PFAS removal efficiency (%) by BBC and Zn-BBC; Figure S5. Pseudo-first-order and pseudo-second-order models for PFAS adsorption onto Zn-BBC; Figure S6. Freundlich, Langmuir and Sips models for adsorption of PFAS onto Zn-BBC; Figure S7. Cl 2p and F 1s spectra for Zn-BBC and Zn-BBC-PFAS revealed by XPS spectra; Table S1. Analyte formula, manufacturer, acronym, and optimum LC-MS/MS parameters for multiple reaction monitoring (MRM) acquisition conditions of the ten target PFASs; Table S2. Chemical structures and p*K_a_* of the ten target PFASs; Table S3. Mobile phase gradient programs; Table S4. MDL and MQL of PFASs in the sample solution at pH7 (*n* = 3); Table S5. Physicochemical properties of banana peel-derived biochar (BBC) and ZnCl_2_-modified banana peel-derived biochar (Zn-BBC); Table S6. Comparison of the adsorptive removal of PFAS between the adsorbent in the present study and those reported in the literature. Table S7. Kinetic parameters of the pseudo-first-order and pseudo-second-order for adsorption of PFASs onto Zn-BBC; Table S8. Adsorption isotherms parameters of PFASs onto Zn-BBC (pH = 7–8) [12,45,71,72,73,74,75,76].

## Abbreviations

The following abbreviations are used in this manuscript:

PFAS	Per- and polyfluoroalkyl substances
FTIR	Fourier transform infrared spectroscopy
XPS	X-ray photoelectron spectroscopy
XRD	X-ray powder diffraction
SEM	scanning electron microscopy
EDS	energy dispersive X-ray spectroscopy
BET	Brunauer–Emmett–Teller
