# Supplementary material for: A Novel Graphitic Biochar Derived from Banana Peels for Efficient PFAS Removal: Mechanistic Insight from Integrated Experiments and DFT Calculations
Source: Toxics. 2026 Feb 27;14(3):204. doi: 10.3390/toxics14030204 (PMC13029874; doi:10.3390/toxics14030204)
Supplement: Supplementary file 1 [file toxics-14-00204-s001.zip › toxics-4137587-supplementary.pdf]

Supplementary Material

A novel graphitic biochar derived from banana peels for efficient PFAS removal: Mechanistic insight from integrated experiments and DFT calculations

Liu-Yi Wei [1,2,^], Ru-Meng Wu [2,^], Zhen-Zhu Liu [1], Feng-Jiao Peng [3,4], Jun-Jie Hu [2], Chang-Gui Pan [2,*]

[1] School of Resources, Environment and Materials, Guangxi University, Nanning 530004, China

[2] Guangxi Laboratory on the Study of Coral Reefs in the South China Sea, School of Marine Sciences, Guangxi University, Nanning 530004, China

[3] Guangdong Provincial Key Laboratory of Chemical Pollution and Environmental Safety & MOE Key Laboratory of Theoretical Chemistry of Environment, SCNU Environmental Research Institute, South China Normal University, Guangzhou 510006, China

[4] School of Environment, South China Normal University, Guangzhou 510006, China

* Corresponding author.

Email address: panchanggui@gxu.edu.cn (Chang-Gui Pan).

^ These authors contributed equally to this work

Number of pages: 21

Number of texts: 3

Number of tables: 8

Number of figures: 7

Contents:

Text S1. Standards and reagents

Text S2. Biochar characterization.

Text S3. Data analysis.

Table S1. Analyte formula, manufacturer, acronym, and optimum LC-MS/MS parameters for multiple reaction monitoring (MRM) acquisition conditions of the ten target PFASs.

Table S2. Chemical structures and pK_a of the ten target PFASs.

Table S3. Mobile phase gradient programs.

Table S4. MDL and MQL of PFASs in the sample solution at pH7 (N=3).

Table S5. Physicochemical properties of banana peel-derived biochar (BBC) and $ZnCl_2$-modified banana peel-derived biochar (Zn-BBC).

Table S6. Comparison of the adsorptive removal of PFAS between the adsorbent in the present study and those reported in the literature

Table S7. Kinetic parameters of the pseudo-first-order and pseudo-second-order for adsorption of PFASs onto Zn-BBC.

Table S8. Adsorption isotherms parameters of PFASs onto Zn-BBC (pH = 7~8).

Figure S1. Preparation flow chart for banana peel-derived biochar (BBC) and $ZnCl_2$-modified banana peel-derived biochar (Zn-BBC).

Figure S2. N_2 adsorption-desorption isotherms for BBC (a) and Zn-BBC (b); Pore size analysis of BBC (c) and Zn-BBC (d).

Figure S3. Zn 2p of Zn-BBC revealed by XPS spectra.

Figure S4. PFAS removal efficiency (%) by BBC and Zn-BBC.

Figure S5. Pseudo-first-order and pseudo-second-order models for PFAS adsorption onto Zn-BBC.

Figure S6. Freundlich, Langmuir and Sips models for adsorption of PFAS onto Zn-BBC.

Figure S7. Cl 2p and F 1s spectra for Zn-BBC and Zn-BBC-PFAS revealed by XPS spectra.

References

Text S1. Standards and reagents.

High-performance liquid chromatography (HPLC)-grade methanol was bought from CNW (Shanghai, China), while liquid chromatograph-mass spectrometer (LC-MS)-grade methanol was obtained from Merck Corporation (Darmstadt, Germany). Zinc chloride ($ZnCl_2$), calcium chloride ($CaCl_2$), magnesium chloride ($MgCl_2$), sodium carbonate (Na_2CO_3), sodium sulfate (Na_2SO_4), sodium hydroxide (NaOH), and hydrochloric acid (HCl) were all obtained from CNW (Shanghai, China). Humic acid (HA) was purchased from Alfa Aesar (Shanghai, China), and LC-MS-grade ammonium acetate was provided by Aladdin (Shanghai, China). Ultrapure water was generated using the Millipore purification system (Millipore, USA). Stock solutions of individual PFASs (1000 ppm) were prepared in methanol and stored in polyethylene (PP) bottles at -18 °C.

Text S2. Biochar characterization.

The physicochemical properties of both BBC and Zn-BBC were characterized using a series of analytical techniques. Scanning electron microscopy-energy dispersive spectroscopy (SEM-EDS, HITACHI SU5000, Japan) was employed to reveal surface morphology and elemental composition. Surface area, pore size, and pore volume were carried out through N_2 adsorption/desorption isotherms using the Brunauer-Emmet-Teller (BET) (Quantachrome Autosorb-iQ USA). Phase composition and lattice parameters were investigated by X-ray diffraction (XRD, Bruker D8 Discover, Germany), with image analysis performed using MDI Jade 6. The Raman spectrum was obtained by Raman spectrometer to detect the structure of the biochar (Raman, Renishaw inVia, UK). Functional groups were characterized using Fourier-transform infrared spectroscopy (FTIR, SHIMADZU IRTrace-100, Japan). Chemical states were determined using X-ray photoelectron spectroscopy (XPS, Thermo Fisher Nexsa, USA), and spectral deconvolution was conducted using Thermo Advantage software. Zeta potential was determined using a Malvern Zetasizer (Mastersizer 3000, UK).

Text S3. Data analysis.

The adsorption equilibrium amount (Q_e) was calculated based on the following equation:

$$Q_e = \frac{(C_0 - C_e)V}{m} \quad \text{(S1)}$$

The formula for calculating the removal efficiency is as follows:

$$R = \frac{(C_0 - C_e)}{C_0} \times 100\% \quad \text{(S2)}$$

where C_0 and C_e (μg/L) represent the concentrations of PFASs at the initial time and t (min), respectively. The parameter m (g) stands for the quantity of BC utilized in the solution, while V (L) denotes the volume of the solution.

The pseudo-first-order (PFO) and pseudo-second-order (PSO) adsorption kinetics models were applied using the following equation:

$$\text{PFO: } Q_t = Q_e(1 - e^{-k_1 t}) \quad \text{(S3)}$$

$$\text{PSO: } \frac{t}{Q_t} = \frac{1}{K_2 Q_e{}^2} + \frac{1}{Q_e} = \frac{1}{V_0} + \frac{t}{Q_e} \quad \text{(S4)}$$

where Q_e and Q_t are the amounts of PFASs adsorbed on adsorbent (μg/g) at equilibrium and at time t (min), respectively; k_1 and k_2 are the adsorption efficiency constants (min^{-1}) for PFO and PSO, respectively. V_0 is the initial adsorption efficiency (μg/g/min).

The adsorption isotherm is represented as follows:

$$\text{Freundlich model: } q_e = K_F C_e{}^{1/n} \quad \text{(S5)}$$

$$\text{Langmuir model: } q_e = \frac{q_m K_L C_e}{1 + K_L C_e} \quad \text{(S6)}$$

$$\text{Sips model: } q_e = \frac{q_m (K_s C_e)^{1/\gamma}}{1 + (K_s C_e)^{1/\gamma}} \quad \text{(S7)}$$

where C_e represents the equilibrium concentration (mg/L) of PFASs in an aqueous solution; q_e is the equilibrium amount (μg/g); K_F ($\mu g^{1-1/n} \cdot L^{1/n} \cdot g^{-1}$) and K_L (L/μg) are the Freundlich and Langmuir sorption constants, respectively. q_m is the maximum adsorption capacity (μg/g); 1/n indicates the degree of non-homogeneity of adsorption. K_s (L/μg) is the Sips model constant and m is the Sips model exponent.

Table S1. Analyte formula, manufacturer, acronym, and optimum LC-MS/MS parameters for multiple reaction monitoring (MRM) acquisition conditions of the ten target PFASs.

Compound	Acronym	Formula	MS/MS mass transition	Fragmentor(V)	Collision energy(V)	Manufacturer
Perfluorobutanoic acid	PFBA	C_3F_7COOH	213.0-168.8[a]	65.00	5.000	Accustandard, USA
Perfluorobutane sulfonic acid	PFBS	$C_4F_9SO_3H$	299.0-79.90[a]/98.90[b]	145.0	41.00/29.00	Wellington Laboratories, Canada
Perfluorohexanoic acid	PFHxA	$C_5F_{11}COOH$	313.0-118.9[a]/268.8[b]	70.00	1.000/13.00	Wellington Laboratories, Canada
Hexafluoropropylene oxide dimer acid	GenX	$C_3F_7OCF(CF_3)COOH$	329.0-285.0 [a]/169.0 [b]	39.00	1.000/11.00	Wellington Laboratories, Canada
Perfluoroheptanoic acid	PFHpA	$C_6F_{13}COOH$	363.0-168.8[a]/318.8[b]	70.00	1.000/9.000	Wellington Laboratories, Canada
Perfluorohexane sulfonic acid	PFHxS	$C_6F_{13}SO_3H$	398.9-79.90[a]/98.90[b]	165.0	53.00/37.00	Wellington laboratories, Canada
6:2 Fluorotonated carboxylic acid	6:2 FTCA	$CF_3(CF_2)_5CH_2COOH$	377.0-293.0[a]	35.0	10.00/3.00	International Laboratory, USA
6:2 Fluorotelomer sulfonate	6:2 FTSA	$CF_3(CF_2)_5(CH_2)_2SO_3H$	427.0-407.0 [a]/81.00 [b]	135.0	24.00/43.00	International Laboratory, USA
Perfluorooctanoic acid	PFOA	$C_7F_{15}COOH$	413.0-368.7[a]/168.9[b]	80.00	1.000/9.000	Accustandard, USA
Perfluorooctane sulfonic acid	PFOS	$C_8F_{17}SO_3H$	498.9-79.90[a]/98.90[b]	190.0	69.00/45.00	Accustandard, USA

[a]Product ion used for quantification.

[b]Product ion used for qualification.

Table S2. Chemical structures and pK_a of the ten target PFASs.

**Chemical**	**pK_a**	**Chemical structure**
PFBA	0.4[a]	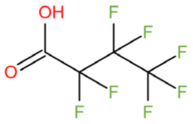
PFBS	0.14[a]	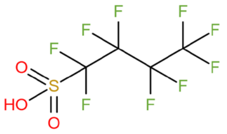
PFHxA	-0.16[a]	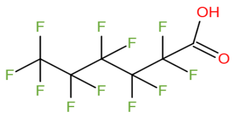
GenX	0.06[b]	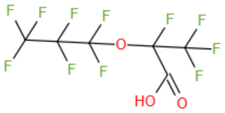
PFHpA	-0.19[a]	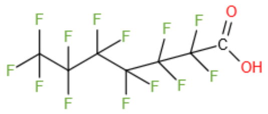
PFHxS	0.14[a]	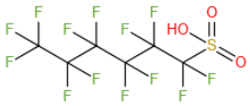
6:2 FTCA	2.82[b]	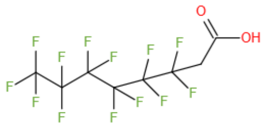
6:2 FTSA	< 1[c]	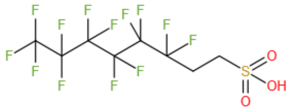
PFOA	-0.2[a]	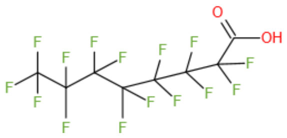
PFOS	-3.27[a]	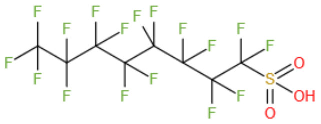

Data are obtained from: [a][1], [b][2], and [c] [3].

Table S3. Mobile phase gradient programs.

Time (minutes)	Mobile phase A (%)	Mobile phase B (%)
0	90.0	10.0
0.1	65.0	35.0
7.0	45.0	55.0
13.0	5.0	95.0
14.0	5.0	95.0
15.0	90.0	10.0

Mobile Phase A: water + 2 mM ammonium acetate.

Mobile Phase B: Methanol.

Table S4. MDL and MQL of PFASs in the sample solution at pH7 (N=3).

Compound	MDL (ng/L)	MQL (ng/L)
PFBA	64.0	213
PFBS	58.0	194
PFHxA	18.0	59.0
GenX	551	1836
PFHpA	48.0	161
PFHxS	149	497
6:2 FTCA	1200	4100
6:2 FTSA	8.0	26.0
PFOA	9.0	30.0
PFOS	275	917

MDL, Method detection limits.

MQL, Method quantification limits.

Table S5. Physicochemical properties of banana peel-derived biochar (BBC) and $ZnCl_2$-modified banana peel-derived biochar (Zn-BBC).

Adsorbent	BET surface area (m^2/g)	Pore volume (cm^3/g)	Pore size (nm)	Particle size (μm)
BBC	34	0.05	3.41	19.33-287.75
Zn-BBC	1157	0.33	3.82	47.16-198.03

Table S6. Comparison of the adsorptive removal of PFAS between the adsorbent in the present study and those reported in the literature.

Adsorbents	PFAS analyzed	Initial concentration (μg/L)	Removal (%)	Equilibrium time	Reference
Fe-doped reed straw-biochar	PFHxA	50	100%	60 min	[4]
	PFBA		88.7%		
Bio–based polyurethane/chitosan	PFHxS	100	55.9%	6 h	[5]
	PFBS		65.1%		
Inorganic silicon-modified activated carbon	PFOA	200	35%	6 h	[6]
	PFOS		75%		
	PFBA		4%		
	PFBS		0%		
Biosolids-sawdust biochar	PFOS	500	97.5%	4 h	[7]
Hydrotalcite calcined at 400 °C	PFOS	100	89%	4 h	[8]
	PFBA		76%		
$ZnCl_2$-modified banana peel biochar	PFBA	100	95.7%	30 min	The present study
	PFBS		100%		
	PFHxA		97.9%		
	GenX		96.5%		
	PFHpA		99.6%		
	PFHxS		100%		
	6:2 FTCA		100%		
	6:2 FTSA		100%		
	PFOA		99.8%		
	PFOS		100%		

Table S7. Kinetic parameters of the pseudo-first-order and pseudo-second-order for adsorption of PFASs onto Zn-BBC.

Compound	PFO					PSO			
	$K_1(h^{-1})$	Q_e(μg/g)	R^2	Q_{exp}	$\|\triangle Q\|$	$K_2(h^{-1})$	Q_e	R^2	$\|\triangle Q\|$
PFBA	0.372	356.24	0.984	407.46	51.22	0.00122	350.82	0.993	56.64
PFBS	0.907	424.69	0.977	432.92	8.23	0.00124	427.81	0.999	5.11
PFHxA	0.647	477.41	0.987	488.79	11.38	0.00787	479.71	0.999	9.08
GenX	0.568	483.47	0.998	497.33	13.86	0.00463	487.39	0.999	9.94
PFHpA	0.747	395.72	0.991	409.55	13.83	0.00819	399.05	0.999	10.50
PFHxS	43.091	381.49	0.989	382.96	1.47	0.03462	381.51	0.999	1.45
6:2 FTCA	0.610	477.59	0.979	485.22	7.63	0.00589	481.11	0.999	4.11
6:2 FTSA	0.652	428.54	0.983	453.87	25.33	0.00690	436.27	0.999	17.60
PFOA	9.3825	451.14	0.995	463.19	12.05	0.01552	453.63	0.999	9.56
PFOS	1.0229	398.43	0.998	401.46	3.03	0.11356	398.55	0.999	2.91

Note: Q_{exp} represents the data derived from the experiment; $|\triangle Q|$ is the difference value between the adsorption capacity fitted by the kinetic model and the experimental value.

Table S8. Adsorption isotherms parameters of PFASs onto Zn-BBC (pH = 7~8).

Compound	Langmuir			Freundlich			Sips			
	q_m	K_L	R^2	1/n	K_F	R^2	q_m	Ks	m	R^2
PFBA	12459.25	0.004	0.62	0.77	86.39	0.52	6073.70	0.019	3.08	0.95
PFBS	5083.13	36.16	0.94	0.33	5080.83	0.72	8056.69	9.37	0.72	0.98
PFHxA	7292.39	0.002	0.99	0.24	7589.79	0.98	4573.27	0.21	6.67	0.96
GenX	7825.17	0.62	0.98	0.28	2436.90	0.99	7834.64	0.35	0.61	0.99
PFHpA	10417.60	0.076	0.96	0.68	1214.06	0.77	7974.06	0.20	1.55	0.97
PFHxS	6218.54	23.04	0.92	0.36	4407.41	0.84	7973.82	7.98	0.75	0.98
6:2 FTCA	5704.76	34.58	0.76	0.34	5535.63	0.45	7322.35	0.17	0.37	0.94
6:2 FTSA	3604.38	148.63	0.86	0.34	4956.04	0.66	9186.50	7.10	0.59	0.98
PFOA	5701.33	30.89	0.91	0.16	7612.58	0.92	9380.28	8.39	0.74	0.93
PFOS	7745.64	13.28	0.96	0.29	5318.93	0.77	9411.25	7.89	0.74	0.99

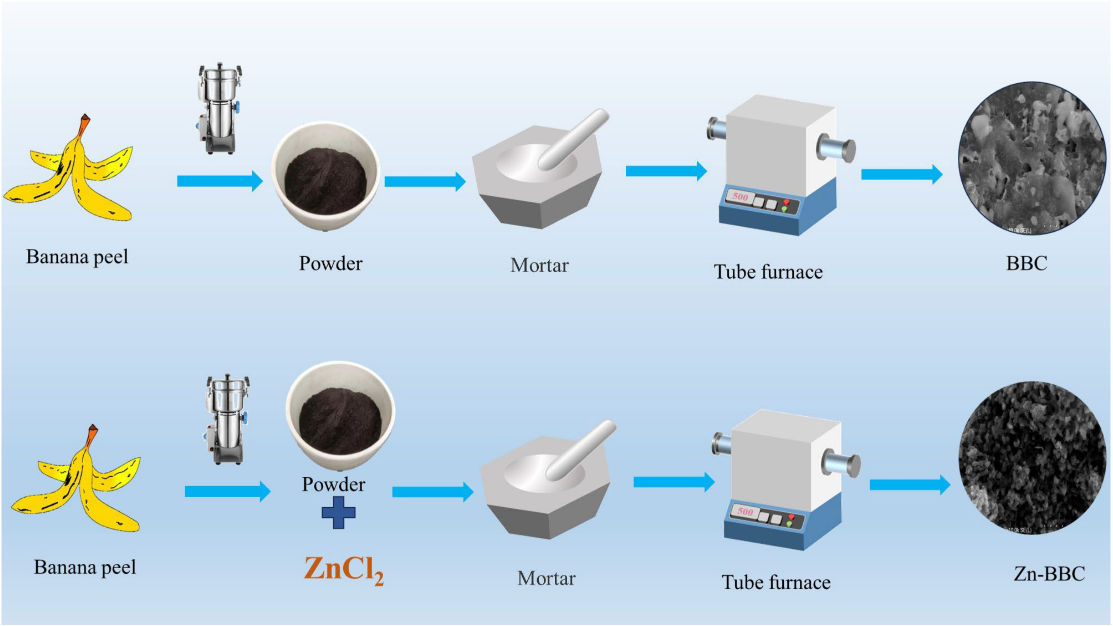

Figure S1. Preparation flow chart for banana peel-derived biochar (BBC) and $ZnCl_2$-modified banana peel-derived biochar (Zn-BBC).

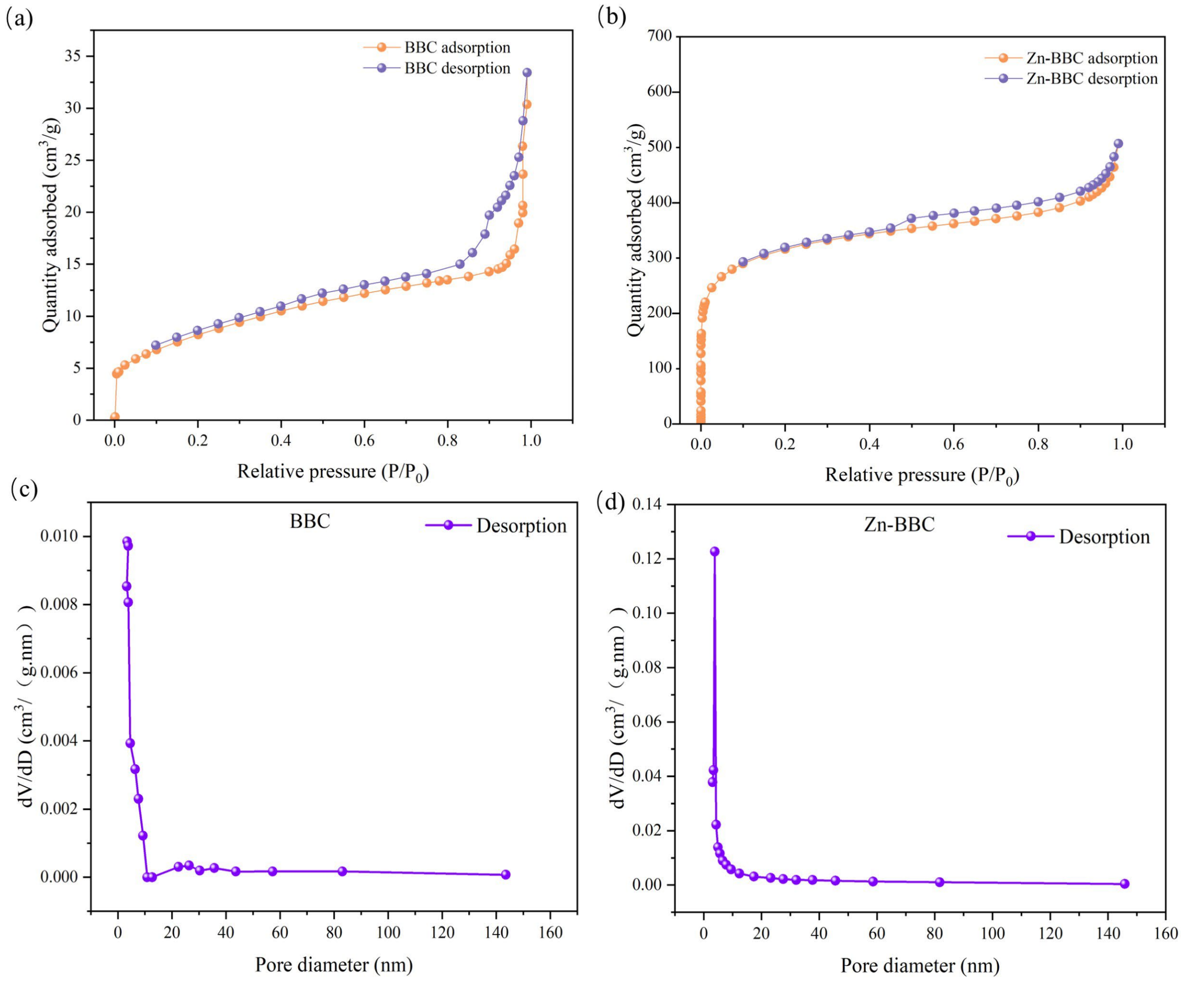

Figure S2. N_2 adsorption-desorption isotherms for BBC (a) and Zn-BBC (b); Pore size analysis of BBC (c) and Zn-BBC (d).

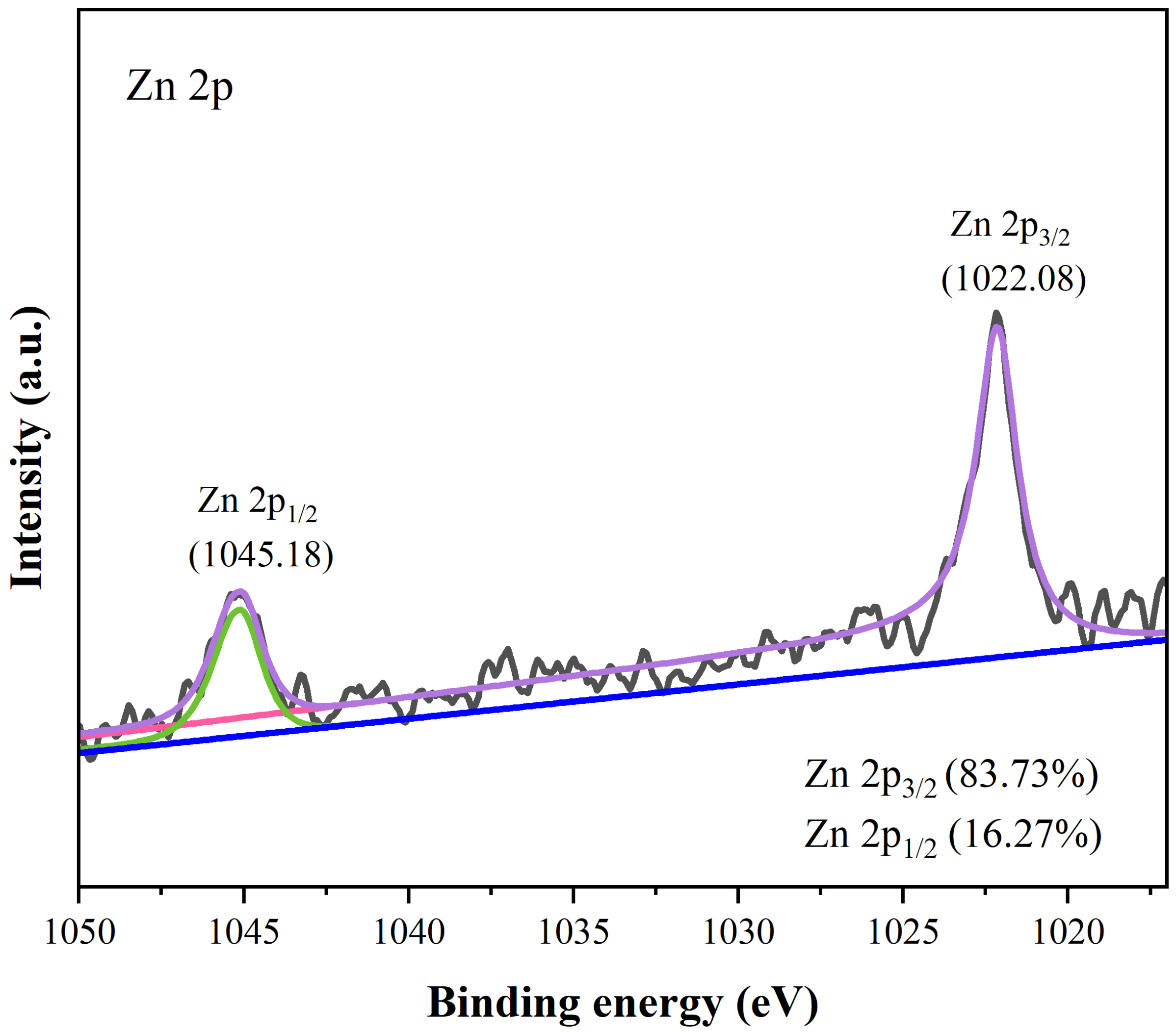

Figure S3. Zn 2p of Zn-BBC revealed by XPS spectra.

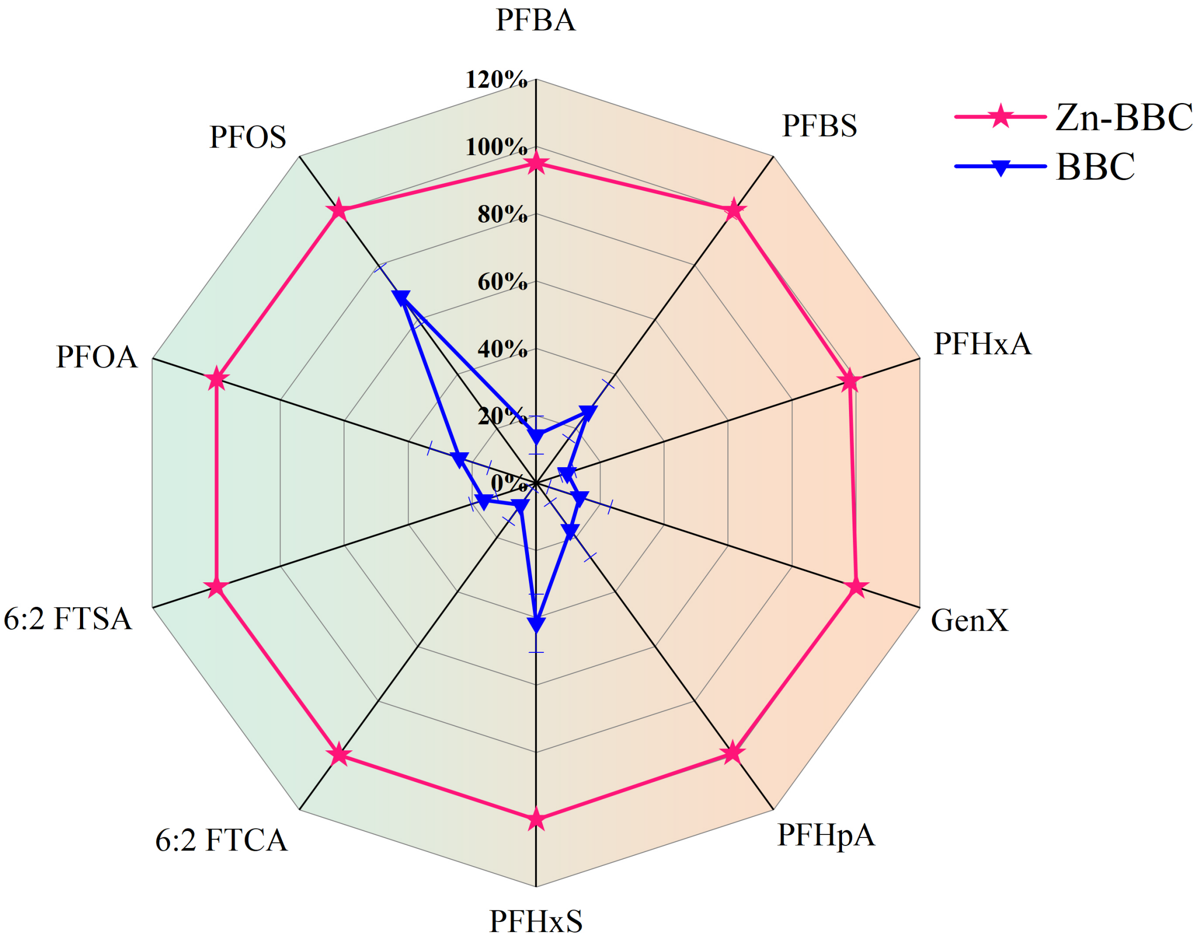

Figure S4. PFAS removal efficiency (%) by BBC and Zn-BBC.

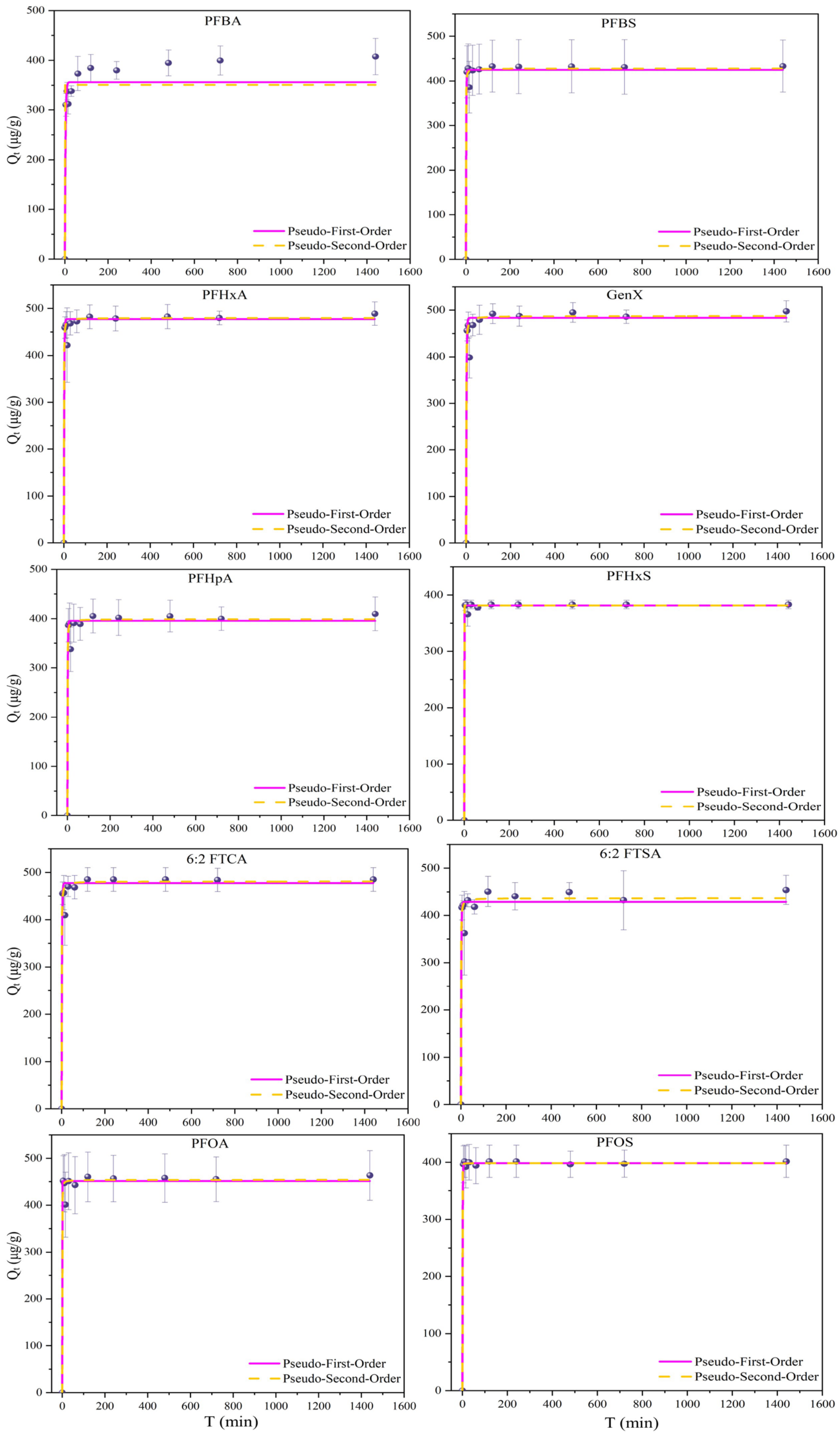

Figure S5. Pseudo-first-order and pseudo-second-order models for PFAS adsorption onto Zn-BBC.

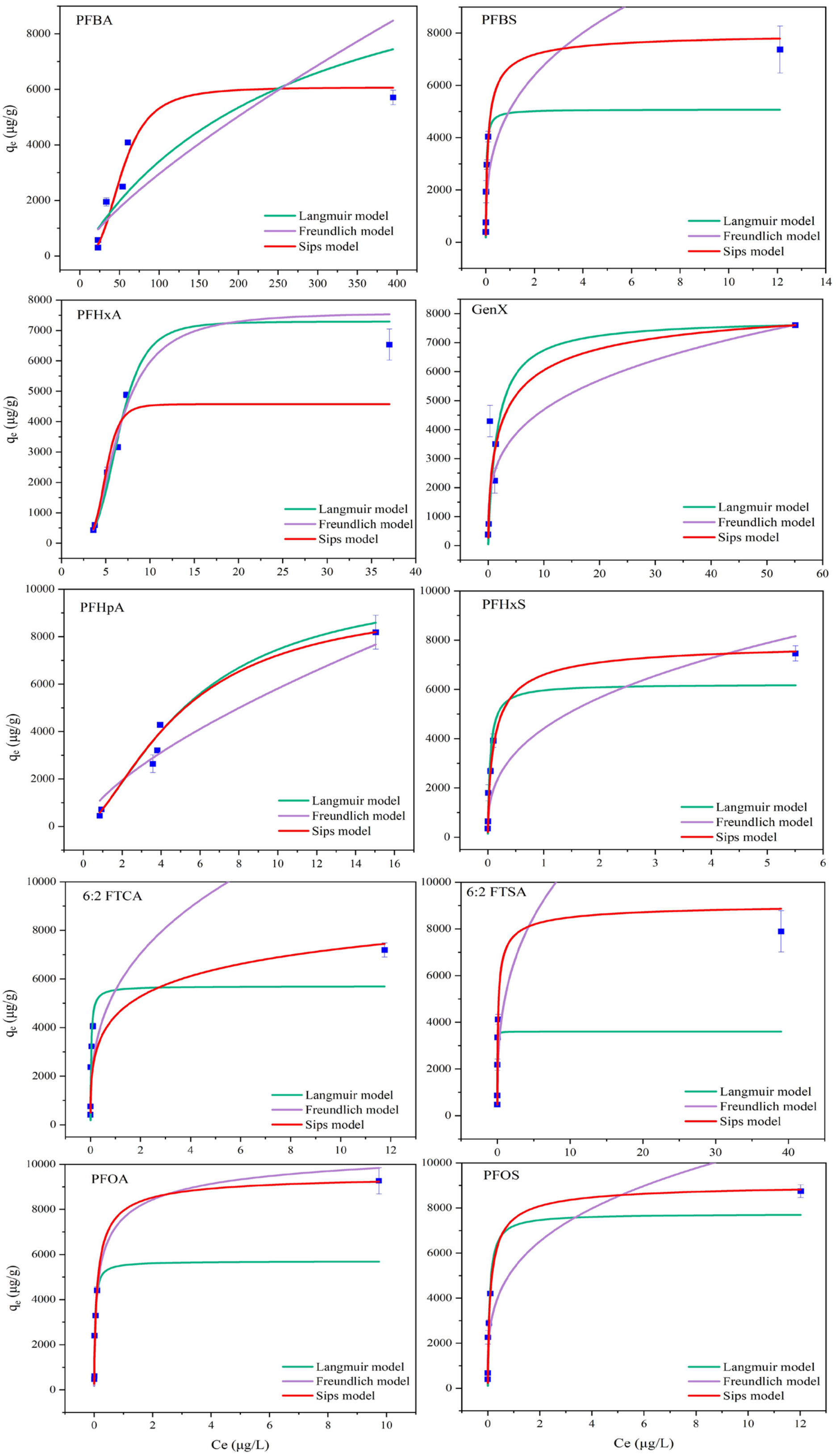

Figure S6. Freundlich, Langmuir and Sips models for adsorption of PFAS onto Zn-BBC.

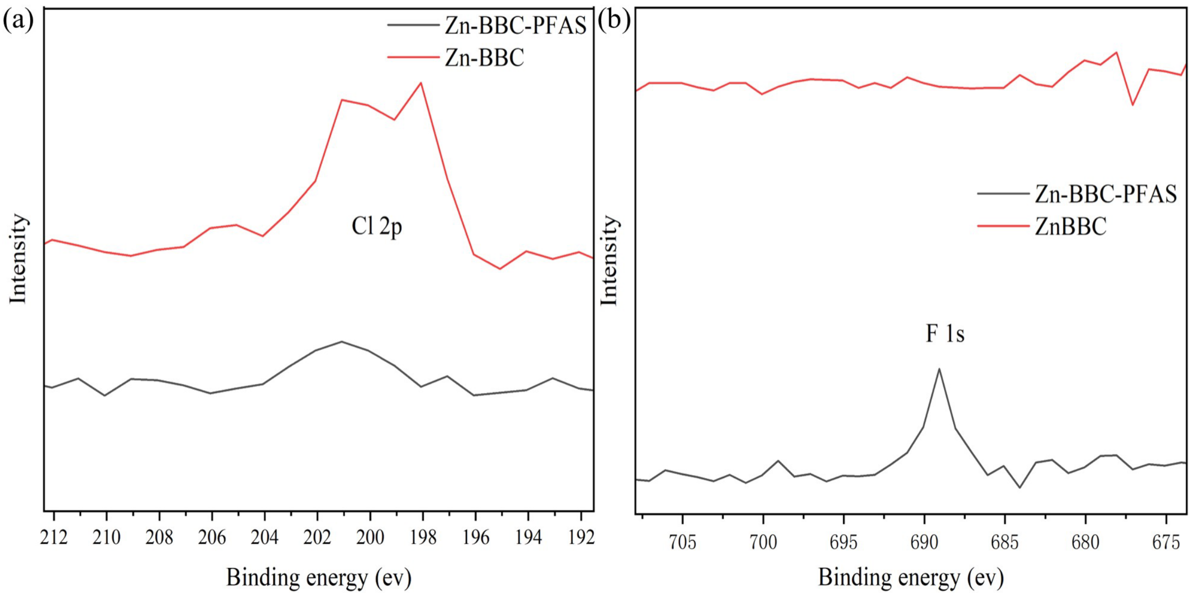

Figure S7. Cl 2p and F 1s spectra for Zn-BBC and Zn-BBC-PFAS revealed by XPS spectra.

References

1. Lei, X., Yao, L., Lian, Q., Zhang, X., Wang, T., Holmes, W., Ding, G., Gang, D.D. and Zappi, M.E. Enhanced adsorption of perfluorooctanoate (PFOA) onto low oxygen content ordered mesoporous carbon (OMC): Adsorption behaviors and mechanisms. *J. Hazard. Mater.* **2022**, *421*, 126810.
2. Gomis, M.I., Wang, Z., Scheringer, M. and Cousins, I.T. A modeling assessment of the physicochemical properties and environmental fate of emerging and novel per- and polyfluoroalkyl substances. *Sci. Total Environ.* **2015**, *505*, 981-991.
3. Tan, H.-M., Pan, C.-G., Yin, C. and Yu, K. Toward systematic understanding of adsorptive removal of legacy and emerging per-and polyfluoroalkyl substances (PFASs) by various activated carbons (ACs). *Environ. Res.* **2023**, *233*, 116495.
4. Liu, N., Li, Y., Zhang, M., Che, N., Song, X., Liu, Y. and Li, C. Efficient adsorption of short-chain perfluoroalkyl substances by pristine and Fe/Cu-loaded reed straw biochars. *Sci. Total Environ.* **2024**, *946*, 174223.
5. Sellaoui, L., Dhaouadi, F., Deghrigue, M., Bouzidi, M., Khmissi, H., Dotto, G.L., Oliveira, M.L.S., Silva, L.F.O., Erto, A., Ernst, B. and Badawi, M. A multilayer adsorption of perfluorohexanesulfonic and perfluorobutanesulfonic acids on bio-based polyurethane/chitosan foam: Advanced interpretation of the adsorption mechanism. *Chem. Eng. J.* **2024**, *489*, 151173.
6. Huang, X., Huang, J., Wang, K., Hao, M., Geng, M., Shi, B. and Hu, C. Comparison of perfluoroalkyl substance adsorption performance by inorganic and organic silicon modified activated carbon. *Water Res.* **2024**, *260*, 121919.
7. Mer, K., Arachchilage, P., Tao, W. and Egiebor, N.O. Activation of sawdust biochar with water and wastewater treatment residuals for sorption of perfluorooctanesulfonic acid in water. *Chemosphere* **2024**, *358*, 142160.
8. Kim, H.-H., Koster van Groos, P.G., Zhao, Y. and Pham, A.L.-T. Removal of PFAS by hydrotalcite: Adsorption mechanisms, effect of adsorbent aging, and thermal regeneration. *Water Res.* **2024**, *260*, 121925.
